# A Genotype‐Independent Transformation and Gene‐Editing System for *Populus*

**DOI:** 10.1111/pbi.70364

**Published:** 2025-09-13

**Authors:** Jiaxing Zhang, Xun Tang, Mengqi Sun, Chang Liu, Hasi Yu

**Affiliations:** ^1^ State Key Laboratory of Tree Genetics and Breeding Northeast Forestry University Harbin China; ^2^ The Center for Basic Forestry Research, College of Forestry Northeast Forestry University Harbin China

**Keywords:** CRISPR/Cas9, gene editing, genotype‐independent transformation, *Populus*, *Rhizobium rhizogenes*

With their rapid growth and broad distribution, *Populus* species contribute significantly to the timber industry, ecological restoration, and bioenergy development (Thakur et al. 2021). The availability of whole‐genome sequences has established *Populus* as a model system for functional genomics and molecular breeding in woody plants (Taylor 2002). Breeding strategies increasingly incorporate transgenic and gene‐editing technologies to enhance key traits such as growth rate, stress tolerance, and wood quality (Zhao et al. 2022). In selecting *Populus* species for genetic modification, adherence to the principle of “the right tree for the right place” is essential, underscoring the importance of aligning genetic material with local environmental conditions to ensure long‐term plantation success (Matheson and Cotterill 1990). However, species‐specific transformation protocols are often difficult to establish and time‐intensive to optimise. Thus, a streamlined, genotype‐independent transformation approach is urgently required to accelerate genetic improvement across *Populus* species.

To overcome the limitations associated with genotype‐dependent media and transformation techniques (Table S1), we developed a universal system (Figure 1a) based on a *Rhizobium rhizogenes* ‐mediated transformation method, validated in multiple plant species (Table S2) and leveraging the distinctive root‐sprouting capacity of poplar (*Populus* L.) (Figure 1b). The system comprises two principal steps: root induction and shoot regeneration from roots.

**FIGURE 1:**
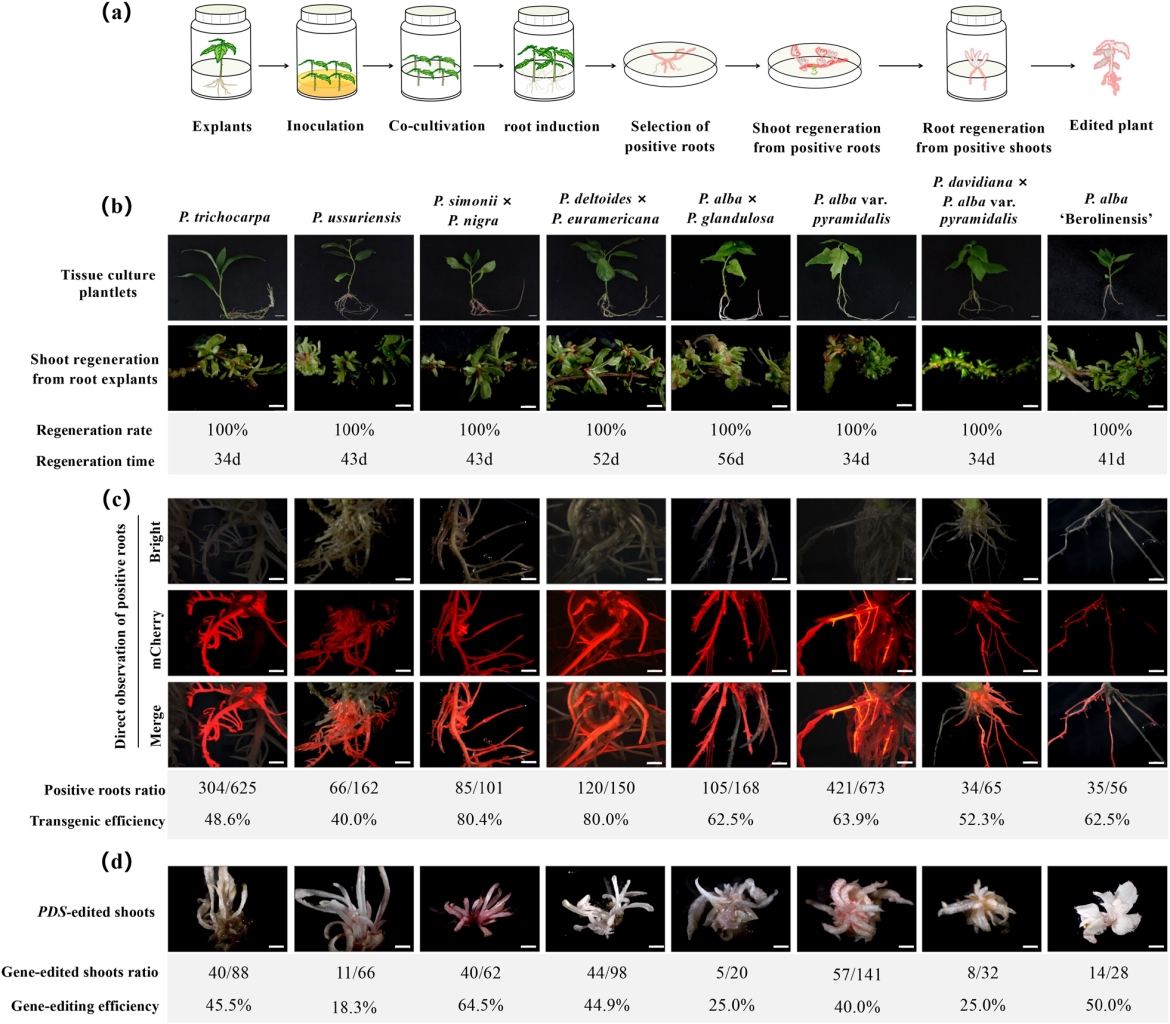
*Rhizobium rhizogenes* ‐mediated transformation and regeneration across multiple *Populus* species. (a) Schematic workflow of *R. rhizogenes* ‐mediated transformation and regeneration in *Populus*. Tissue culture plantlets aged 15–30 days were excised, and the basal cut surfaces were inoculated with *R. rhizogenes* carrying reporter and gene‐editing constructs for 10–15 min. Following 2–3 days of co‐cultivation, explants were transferred to hormone‐free medium to induce root formation. Newly generated roots developed within 14–30 days, after which mCherry‐positive roots (indicated in red) were selected and cultured for shoot regeneration. Transgenic and gene‐edited shoots were obtained within 30–60 days and subsequently transferred to root induction medium. Edited plantlets with established root systems were recovered within 14–30 days. (b) Top: Tissue culture plantlets (15–30 days old) from various *Populus* species. Scale bar: 1 cm. Bottom: *De novo* shoot regeneration from root explants within 2 months on shoot induction medium, with a shoot induction rate approaching 100%. Scale bar: 2 mm. (c) Transgenic roots expressing mCherry visualised in red. Transformation efficiency was determined by calculating the proportion of mCherry‐positive roots relative to the total number of roots, which ranged from 40.0% to 80.4% across eight *Populus* species. Scale bar: 2 mm. (d) PDS‐edited albino *Populus* plants regenerated from root explants on shoot induction medium. Editing efficiency was determined by calculating the proportion of albino shoots among all regenerated shoots, with values ranging from 18.3% to 64.5% across eight *Populus* species. Scale bar: 2 mm.

In the first step, shoots excised from axenic plantlets were immersed at their basal cut ends in an *R. rhizogenes* suspension, followed by co‐cultivation and transfer to a sucrose‐free, hormone‐free rooting medium (Figure S1). To improve rooting efficiency and facilitate the identification of positive transformants, the antibiotic selection system was replaced with fluorescent markers (mCherry or EGFP) (Figure 1c and Figure S2). Eliminating antibiotic selection permits transformants to grow under physiologically normal conditions, preserving developmental potential and reducing unintended selection pressure. In addition, fluorescence‐based selection enables direct visualisation of transgene expression at early stages of root development, shortening the screening period from 4 to 6 weeks to 2 to 4 weeks.

Rooting efficiency was further improved by the use of hormone‐free medium, as earlier studies indicated that excessive auxin application may suppress root regeneration (Yu et al. 2017). The presence of root‐inducing (RolA/B/C) genes within the T‐DNA region of the *R. rhizogenes* Ri plasmid enabled efficient root formation without the need for exogenous auxin (Xiang et al. 2016). In addition, previous reports have shown that roots induced by *R. rhizogenes* often originate from single cells, resulting in a reduced incidence of chimerism in transgenic roots (Roychowdhury et al. 2017). Collectively, these optimizations in the first step enhance the efficiency of transgenic root induction and facilitate rapid enrichment of homozygous or bi‐allelic mutations.

In the second step, *de novo* shoots were regenerated from roots that exhibited uniform, strong fluorescence along their entire length at 14–30 days post‐inoculation. Under natural conditions, root sprouting in perennial woody plants typically occurs only after the roots reach a certain developmental stage (Wiehle et al. 2009). Moreover, the initiation and progression of root sprout primordia require adequate time and favourable physiological conditions; as a result, newly formed roots generally lack the immediate capacity to produce shoots (Wan et al. 2006). To overcome this limitation, thidiazuron (TDZ), a synthetic cytokinin, was incorporated during the shoot regeneration phase. Within 30–60 days, adventitious shoots regenerated successfully from the fluorescent‐positive roots, demonstrating the system's high efficiency (Figure S3). The regenerated shoots elongated and formed adventitious roots upon transfer to the rooting medium, completing the regeneration cycle (Figure S3).

To evaluate the gene‐editing efficiency of the system, the Phytoene desaturase (*PDS*) gene was selected for functional validation. The previously reported T2 sgRNA targeting *PDS* (Fan et al. 2015) was cloned into the custom “1‐step‐Cas9‐ATU6‐EGFP/mCherry” binary vector for transformation (Figure S4). Given the absence of genomic data for certain *Populus* species, the ability of the T2 sgRNA to target *PDS* across all eight tested genotypes was verified through PCR amplification and sequencing (Figure S5). An average editing efficiency of 39.2% was observed across the eight genotypes, highlighting the strong editing capacity of the system (Figure 1d). Further sequence analysis of DNA isolated from albino shoots confirmed successful editing of the *PDS* gene in all eight species (Figure S6). Notably, editing efficiencies varied among genotypes, potentially reflecting differences in genomic architecture or DNA repair pathways. Additional mechanistic studies will be needed to further optimise editing outcomes across diverse genetic backgrounds.

This study presents a robust *R. rhizogenes* ‐mediated transformation system for *Populus*, combining fluorescent selection with a simplified two‐medium regeneration protocol (Appendix S1). The approach markedly shortens the transformation cycle, improves efficiency, and performs reliably across diverse genotypes, highlighting its adaptability. Its simplicity and low cost establish a broadly applicable platform for transgenic production and genome editing in *Populus*, with potential for extension to other woody species. The system provides a practical method for advancing both fundamental research and applied breeding in forest trees.

## Author Contributions

H.Y. and C.L. conceived and supervised the experiments. J.Z. and X.T. designed the experiments. J.Z. and M.S. carried out the experiments. J.Z., X.T., H.Y., and C.L. wrote the paper. All authors read and approved the content.

## Conflicts of Interest

The authors declare no conflicts of interest.

## Supporting information


**Appendix S1:** Plant materials, methods and map of the 1‐step‐Cas9‐AtU6‐EGFP/mCherry vector.

**Figure S1:** Root induction in a representative *Populus* genotype.
**Figure S2:** EGFP expression in newly generated roots of various *Populus* species.
**Figure S3:** Shoot regeneration from transgenic roots in a representative *Populus* genotype.
**Figure S4:** Construction of the *PDS*‐targeting knockout vector for *Populus*.
**Figure S5:** Detection of single nucleotide polymorphisms at the T2 sgRNA target site in the *PDS* gene.
**Figure S6:** Gene editing and sequencing analysis of the *Populus PDS* gene.

**Table S1:** Summary of Agrobacterium‐mediated transformation protocols in *Populus*.

**Table S2:** Summary of recent literature on *Rhizobium rhizogenes* ‐mediated plant transformation.

## Data Availability

The data that supports the findings of this study are available in the Supporting Information of this article.
