# Supplementary material for: A Genotype‐Independent Transformation and Gene‐Editing System for Populus
Source: Plant Biotechnol J. 2025 Sep 13;24(2):520–2. doi: 10.1111/pbi.70364 (PMC12906817; doi:10.1111/pbi.70364)
Supplement: Supplementary file 1 — Appendix S1: Plant materials, methods and map of the 1‐step‐Cas9‐AtU6‐EGFP/mCherry vector. [file PBI-24-520-s001.docx]

**Plant materials**

The shoots were cut from the tissue-cultured sterile plantlets of eight poplar germplasms: *Populus trichocarpa*, *Populus ussuriensis*, *Populus simonii* *× Populus nigra*, *Populus deltoides × Populus euramericana* ‘Nanlin 895,’ *Populus alba × Populus glandulosa* ‘84K,’ *Populus davidiana* × *P. alba* var. pyramidalis*, P. alba* var. pyramidalis, and *Populus alba* ‘Berolinensis.’ The plants were cultivated in a growing chamber under the following conditions: temperature 22 ± 1°C, relative humidity 35 ± 5%, light intensity 10,000 Lux, and a photoperiod of 16 h light/8 h dark.

Under sterile conditions, the apical young buds (approximately 3 cm in length) of the tissue-cultured plantlets were excised using a sterilized scalpel and then transferred to rooting medium for rooting. The obtained plantlets were used for subsequent experiments.

**Reagents**

**Vectors**

1-step-Cas9-AtU6-EGFP (based on pCAMBIA1300, custom-constructed)

1-step-Cas9-AtU6-mCherry (based on pCAMBIA1300, custom-constructed)

**Strains**

*Rhizobium rhizogenes* (K599)

**Antibiotics**

Kanamycin (Kan) (Cat. #60615; Sigma-Aldrich, St. Louis, MO, USA)

Streptomycin (Str) (Cat. #T8902; Sigma-Aldrich)

Cefotaxime (Cef) (Cat. #C7039; Sigma-Aldrich)

**Culture Media**

Tryptone (Cat. #211705, BD Difco; BD Biosciences, Franklin Lakes, NJ, USA)

Yeast Extract (Cat. #212750, BD Difco; BD Biosciences)

Sodium Chloride (NaCl) (Cat. #S9888; Sigma-Aldrich)

Agar (Cat. #A1296; Sigma-Aldrich)

WPM Basal Medium (Cat. #M519; PhytoTech Labs, Lenexa, KS, USA)

Sucrose (Cat. #S7903; Sigma-Aldrich)

MES Buffer (Cat. #M3671; Sigma-Aldrich)

Acetosyringone (As) (Cat. #D134406; Sigma-Aldrich)

**Plant Hormones**

Thidiazuron (TDZ) (Cat. #P6186; Sigma-Aldrich)

**Instruments**

Electric Thermostatic Drying Oven (Model: HWL-125; Tianjin Laboratory Instrument Equipment Co., Ltd., Tianjin, China)

Vertical Constant Temperature Shaking Incubator (Model: ZQPL-200; Tianjin Laboratory Instrument Equipment Co., Ltd.)

Vertical Automatic Pressure Steam Sterilizer (Model: GI54DWS; Zhiwei Xiamen Instrument Co., Ltd., Xiamen, China)

Clean Bench (Model: DL-CJ-2NDⅠ; Beijing Donglian Haer Instrument Manufacturing Co., Ltd., Beijing, China)

RDN Artificial Climate Chamber (Model: RDN-1000D-N; Ningbo Southeast Instrument Co., Ltd., Ningbo, China)

Stereomicroscope (Model: Discovery.V20; Carl Zeiss, Oberkochen, Germany)

**Medium Preparation**

- LB Medium was prepared using 10 g L⁻¹ tryptone, 5 g L⁻¹ yeast extract, 5 g L⁻¹ NaCl, and 15 g L⁻¹ agar.
- Poplar Co-Cultivation Medium (CM) was prepared using 2.41 g L⁻¹ WPM, 25 g L⁻¹ sucrose, 0.5 g L⁻¹ MES, and 6 g L⁻¹ agar at a pH of 5.8. After sterilization, 100 μM As was added.
- Poplar Rooting Medium (RM) was prepared using 2.41 g L⁻¹ WPM, 25 g L⁻¹ sucrose, 0.5 g L⁻¹ MES, and 6 g L⁻¹ agar at a pH of 5.8. After sterilization, 400 mg L⁻¹ Cef was added.
- Poplar Shoot Induction Medium (SIM) was prepared using 2.41 g L⁻¹ WPM, 25 g L⁻¹ sucrose, 0.5 g L⁻¹ MES, 0.02 mg L⁻¹ TDZ, 6 g L⁻¹, and agar at a pH of 5.8. After sterilization, 400 mg L⁻¹ Cef was added.

**Procedure**

**1.** **Vector construction**

This study modified the pCAMBIA1300 vector as the backbone by removing the resistance gene using specific enzymes. The enhanced green (EGFP) and red (mCherry) fluorescent protein marker genes were then inserted and placed under the control of the 35S promoter to facilitate the screening and observation of transgenic plants. The modified vectors were designated 1-step-Cas9-AtU6-EGFP and 1-step-Cas9-AtU6-mCherry (Figure S7). See detailed sequence information in Appendixes 1.1 and 1.2.

Based on the reported coding sequence of the *PDS* gene in *Populus tomentosa*, a highly efficient 20-bp target sequence was selected. The PAM sequence (NGG) was positioned downstream of the sgRNA target site. The final selected target site was sgRNA2 (5′-GTGTTATCAAGGTCCGGTCT-3′) (PAM: TGG) (Fan et al., 2015). Sticky ends matching the BsaⅠ cut site were added to the sgRNA sequence as sgRNA primers (Table S2), which were then annealed by cooling from 98°C to 16°C to form double-stranded DNA with sticky ends. The annealed sgRNA was stored at 4°C for subsequent ligation.

The 1-step-Cas9-AtU6-EGFP/mCherry plasmid was digested with BsaⅠ, followed by incubation in a PCR machine at 37°C for 2 h. After digestion, the enzyme was inactivated at 65°C for 20 min. The linearized vector was then ligated with 200-fold diluted sgRNA using T4 ligase at room temperature for 10 min. The recombinant vector was subsequently transformed into *Escherichia coli* DH5α competent cells for amplification. To verify successful vector construction, DNA sequencing was conducted to ensure the accuracy and integrity of the vector structure.


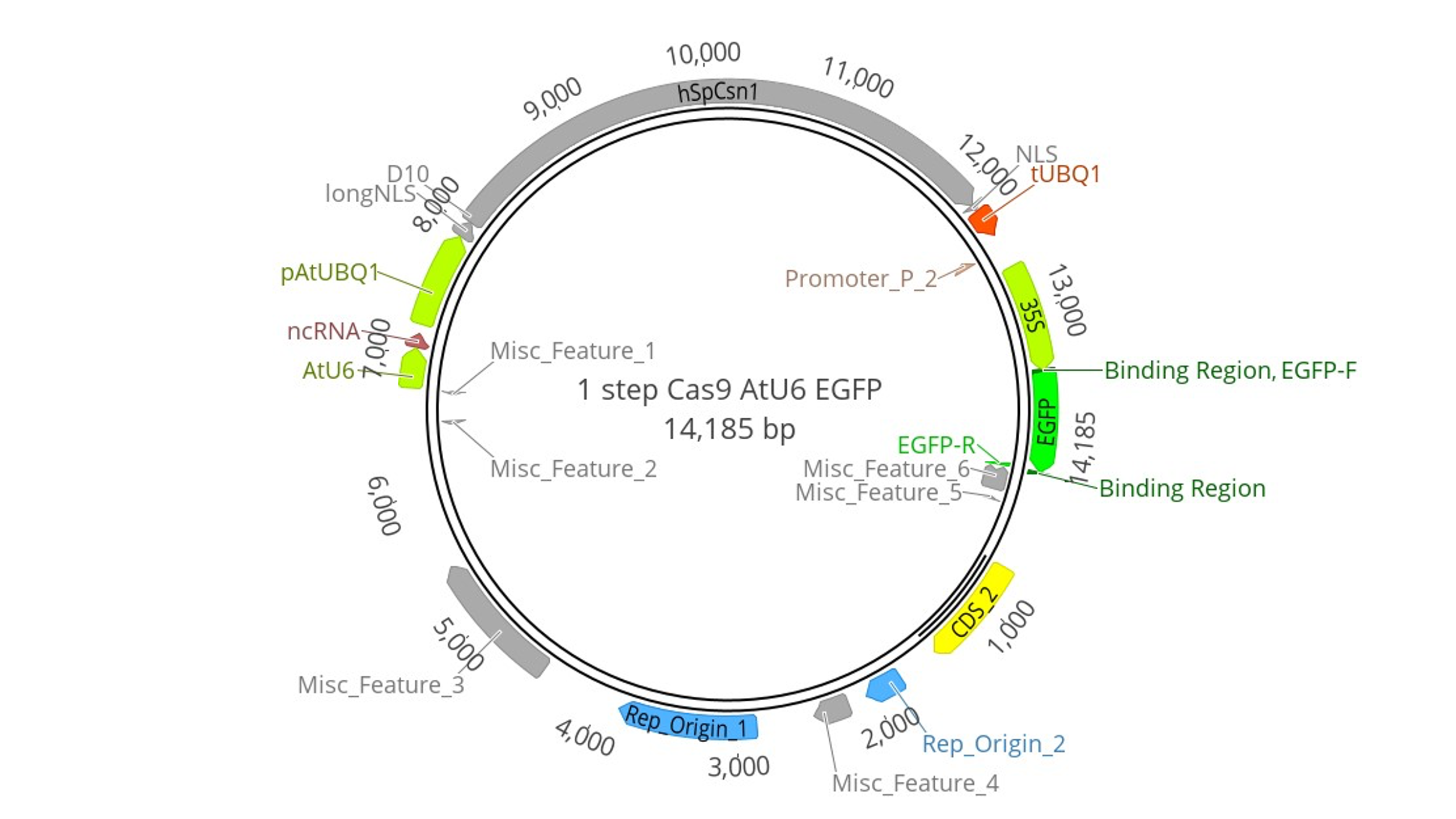


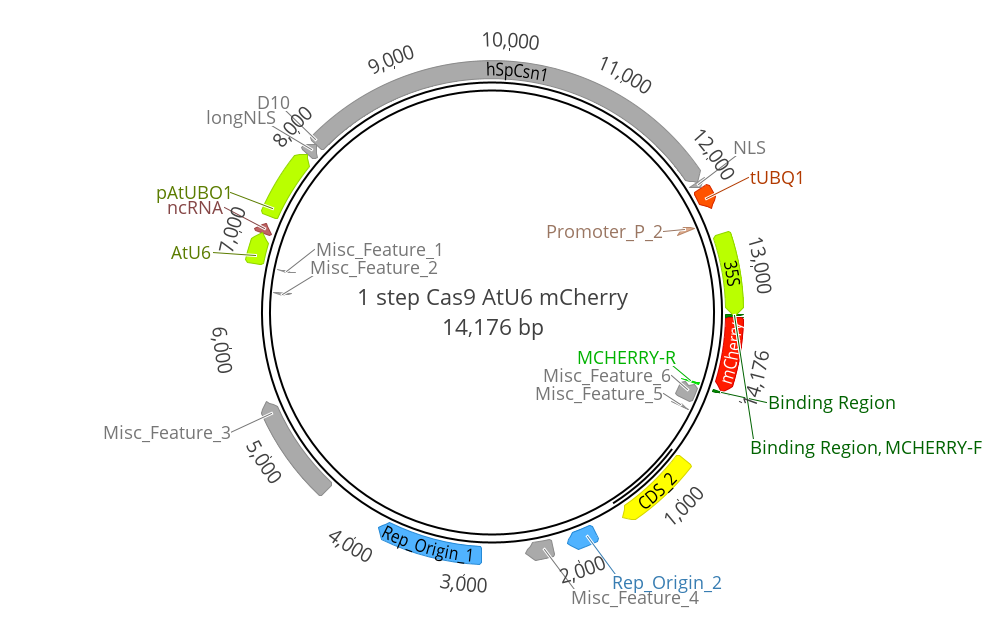


**Figure** **S7**. 1-step-Cas9-AtU6-EGFP/mCherry vector map.

**Table S2**. sgRNA primers used in this study.

| Primer name | Primer sequence（5′–3′） |
| --- | --- |
| T2-F | gattgGTGTTATCAAGGTCCGGTCT |
| T2-R | aaacGCTCAAGTTCAATGCACTCAc |

**2.** ***R. rhizogenes*-induced hairy roots in transgenic poplar trees**

Under sterile conditions, a single colony of *R.rhizogenes* was selected and inoculated into 4 mL LB liquid medium. Next, 4 μL Kan (50 mg/mL) and 4 μL Str (50 mg/mL) were added and cultured overnight at 28°C and shaking at 220 rpm. Thereafter, the culture was transferred to 50 mL LB liquid medium (with antibiotics: 2.5 mg Kan, 2.5 mg Str), continuously cultured until the OD_600_ reached 0.6, and stored for future use.

The 4^th^–5^th^ stem nodes of 2-to-8-week-old *P. trichocarpa*, *P. ussuriensis*, *P. simonii × P. nigra*, *P. deltoides × P. euramericana* ‘Nanlin 895,’ *P. alba × P. glandulosa* ‘84K,’ *P. davidiana* × *P. alba* var. pyramidalis*, P. alba* var. pyramidalis, *P. alba* ‘Berolinensis’ plants were cut under sterile conditions, keeping the apical bud. Afterward, the cut ends with apical buds were immersed in *R. rhizogenes* K599 suspension for 12–15 min, and surface bacteria were removed using sterile filter paper.

The infected stem segments were transferred to a co-culture medium and co-cultured at 25 ± 2°C for 2 days. After co-culture, the samples were washed 3–5 times with sterile water containing cefotaxime (Cef) for 30 min each time and dried with sterile filter paper. Subsequently, the co-cultured poplar stem segments were transferred to a rooting medium without sucrose—a crucial step in preventing the recurrence of *R. rhizogenes*—and cultured under the following culture conditions: 16 h/8 h light/dark cycle, 25±2°C, and 50–60% relative humidity. After 8 days, the stem base swelled (Figure 1b); after 11 days, white adventitious roots appeared; after 30 days, a large number of white roots formed.

**3. Fluorescence signal detection of poplar hairy roots**

To verify the expression of foreign genes in transgenic hairy roots, stereomicroscopy (Carl Zeiss) was used to detect EGFP and mCherry fluorescence. Briefly, after 30 days of hairy root induction, the roots were removed from the medium and rinsed with sterile water to remove residual bacteria and medium. The cleaned samples were transferred to a sterile Petri dish, which was placed on the microscope stage for observation. The fluorescence detection parameters were set as follows: mCherry: excitation wavelength 587 nm, emission wavelength 610 nm; EGFP: excitation wavelength 488 nm, emission wavelength 510 nm. Subsequently, the fluorescence signals were observed to confirm the successful transfer of the foreign vector into the hairy roots. Roots emitting red fluorescence under mCherry or green fluorescence under EGFP were considered transgenic.

**4. Regeneration of transgenic roots to form transgenic plants**

Transgenic hairy roots were aseptically transferred to root induction medium. After approximately 20 days, buds formed from the roots, and after another 20 days, 2–3-cm young shoots developed. Healthy shoots (approximately 2 cm long) were selected and transferred to rooting medium. After 1–2 weeks, complete root systems formed, resulting in fully developed transgenic plants.

**5. Efficiency calculations**

**5.1. Shoot regeneration rate calculation**

The shoot regeneration rate was determined to assess the efficiency of adventitious shoot formation from initial explants. The formula used was as follows:

Shoot regeneration rate (%) = (Number of explants forming adventitious shoots/Total initial explants) × 100%

Explants exhibiting visible shoot formation after a defined culture period were counted.

**5.2. Transgenic efficiency determination**

Transgenic efficiency was evaluated based on the proportion of roots expressing fluorescent markers (mCherry or EGFP) following transformation. The formula applied was as follows:

Transgenic efficiency (%) = ( mCherry or EGFP positive roots/Total screened roots) × 100%

Roots were examined under a fluorescence microscope, and only those with clear and stable reporter gene expression were scored as positive. To account for variability, three biological replicates were used, with at least 100 roots screened per replicate.

**5.3. Editing efficiency analysis**

The editing efficiency was calculated to quantify the success of targeted genome modifications in transgenic plants. The formula employed was as follows:

Editing efficiency (%) = (Plants with verified edits/Total transgenic plants analyzed) × 100%

Three biological replicates were used, with a minimum of 20 independent transgenic plants analyzed per replicate. The results were pooled for final efficiency determination.

**Tips:**

1. Ensure thorough removal of *Agrobacterium*.

2. Handle gently to avoid damaging the roots.

**References**

Fan, D, Liu T, Li C, Jiao B, Li S, Hou Y, Luo K (2015) Efficient CRISPR/Cas9-mediated targeted mutagenesis in Populus in the first generation. *Scientific Reports* **5**: 12217.

**1-step Cas9 AtU6 EGFP vector Genbank sequence, including the metadata and annotations.**

LOCUS 1_step_Cas9_AtU6 14185 bp DNA circular UNA 03-MAR-2025

DEFINITION Gibson Assembly of PSC1-BplU3-4 - BlpR=PCR 产物 into Digestion of

pCAMBIA1300-psgR (modified)= Xhol.

ACCESSION urn.local...a5-ivpuhmw

VERSION urn.local...a5-ivpuhmw

KEYWORDS .

SOURCE

ORGANISM

.

FEATURES Location/Qualifiers

misc_feature complement(<14167..167)

/label="Misc_Feature_6"

misc_feature complement(234..259)

/label="Misc_Feature_5"

CDS 684..1478

/label="CDS_2"

rep_origin 1769..2049

/label="Rep_Origin_2"

misc_feature 2189..2449

/label="Misc_Feature_4"

rep_origin 2859..3859

/label="Rep_Origin_1"

misc_difference 3026

/label="T to A"

misc_feature 4452..5452

/label="Misc_Feature_3"

misc_feature complement(6493..6518)

/label="Misc_Feature_2"

misc_feature complement(6738..>6760)

/label="Misc_Feature_1"

promoter 6766..7058

/label="AtU6"

ncRNA 7059..7156

/label="ncRNA"

promoter 7228..7907

/label="pAtUBQ1"

misc_feature 7908..8027

/note="longNLS"

/label="longNLS"

misc_feature 8028..12128

/note="hSpCsn1"

/modified_by="User"

/label="hSpCsn1"

misc_feature 8052..8054

/note="D10"

/label="D10"

misc_feature 12129..12176

/note="NLS"

/label="NLS"

terminator 12186..12393

/label="tUBQ1"

origin 12406^12407

/label="origin"

misc_feature complement(12453..12507)

/label="Promoter_P_2"

/note="Geneious type: promoter prokaryotic"

misc_feature 12655..13435

/label="Promoter_P_1"

/note="Geneious type: promoter prokaryotic"

ligation 13418..13436

/label="Ligation"

primer_bind 13418..13441

/Sequence="CTCTACAAATCTATCTCTCTCGAG"

/Hairpin_Tm="38.7"

/Tm="55.5"

/Self_Dimer_Tm="5.9"

/%GC="41.7"

/modified_by="User"

/label="Binding Region"

primer_bind 13442..13459

/Sequence="ATGGTGAGCAAGGGCGAG"

/Hairpin_Tm="47.5"

/Tm="59.7"

/Self_Dimer_Tm="None"

/%GC="61.1"

/created_by="primer3"

/modified_by="User"

/label="EGFP-F"

primer_bind complement(14140..14161)

/Sequence="TTACTTGTACAGCTCGTCCATG"

/Hairpin_Tm="None"

/Tm="58.2"

/Self_Dimer_Tm="None"

/%GC="45.5"

/created_by="primer3"

/modified_by="User"

/label="EGFP-R"

misc_feature <14162..14185

/label="PSC1-BplU3-4 - BlpR=PCR 产物"

/note="Geneious type: Inserted sequence"

primer_bind 14162..14185

/created_by="primer3"

/annotation_group="441838253606200dd92c503-e200-44ee-9a5c-

4f9bc535cbd5"

/%GC="37.5"

/Tm="55.5"

/Hairpin_Tm="None"

/Self_Dimer_Tm="3.6"

/Sequence="CTCGAGTTTCTCCATAATAATGTG"

/label="Binding Region"

ligation 14167..14185

/label="Ligation"

ORIGIN

1 tgagtagttc ccagataagg gaattagggt tcctataggg tttcgctcat gtgttgagca

61 tataagaaac ccttagtatg tatttgtatt tgtaaaatac ttctatcaat aaaatttcta

121 attcctaaaa ccaaaatcca gtactaaaat ccagatcccc cgaattaatt cggcgttaat

181 tcagtacatt aaaaacgtcc gcaatgtgtt attaagttgt ctaagcgtca atttgtttac

241 accacaatat atcctgccac cagccagcca acagctcccc gaccggcagc tcggcacaaa

301 atcaccactc gatacaggca gcccatcagt ccgggacggc gtcagcggga gagccgttgt

361 aaggcggcag actttgctca tgttaccgat gctattcgga agaacggcaa ctaagctgcc

421 gggtttgaaa cacggatgat ctcgcggagg gtagcatgtt gattgtaacg atgacagagc

481 gttgctgcct gtgatcaccg cggtttcaaa atcggctccg tcgatactat gttatacgcc

541 aactttgaaa acaactttga aaaagctgtt ttctggtatt taaggtttta gaatgcaagg

601 aacagtgaat tggagttcgt cttgttataa ttagcttctt ggggtatctt taaatactgt

661 agaaaagagg aaggaaataa taaatggcta aaatgagaat atcaccggaa ttgaaaaaac

721 tgatcgaaaa ataccgctgc gtaaaagata cggaaggaat gtctcctgct aaggtatata

781 agctggtggg agaaaatgaa aacctatatt taaaaatgac ggacagccgg tataaaggga

841 ccacctatga tgtggaacgg gaaaaggaca tgatgctatg gctggaagga aagctgcctg

901 ttccaaaggt cctgcacttt gaacggcatg atggctggag caatctgctc atgagtgagg

961 ccgatggcgt cctttgctcg gaagagtatg aagatgaaca aagccctgaa aagattatcg

1021 agctgtatgc ggagtgcatc aggctctttc actccatcga catatcggat tgtccctata

1081 cgaatagctt agacagccgc ttagccgaat tggattactt actgaataac gatctggccg

1141 atgtggattg cgaaaactgg gaagaagaca ctccatttaa agatccgcgc gagctgtatg

1201 attttttaaa gacggaaaag cccgaagagg aacttgtctt ttcccacggc gacctgggag

1261 acagcaacat ctttgtgaaa gatggcaaag taagtggctt tattgatctt gggagaagcg

1321 gcagggcgga caagtggtat gacattgcct tctgcgtccg gtcgatcagg gaggatatcg

1381 gggaagaaca gtatgtcgag ctattttttg acttactggg gatcaagcct gattgggaga

1441 aaataaaata ttatatttta ctggatgaat tgttttagta cctagaatgc atgaccaaaa

1501 tcccttaacg tgagttttcg ttccactgag cgtcagaccc cgtagaaaag atcaaaggat

1561 cttcttgaga tccttttttt ctgcgcgtaa tctgctgctt gcaaacaaaa aaaccaccgc

1621 taccagcggt ggtttgtttg ccggatcaag agctaccaac tctttttccg aaggtaactg

1681 gcttcagcag agcgcagata ccaaatactg tccttctagt gtagccgtag ttaggccacc

1741 acttcaagaa ctctgtagca ccgcctacat acctcgctct gctaatcctg ttaccagtgg

1801 ctgctgccag tggcgataag tcgtgtctta ccgggttgga ctcaagacga tagttaccgg

1861 ataaggcgca gcggtcgggc tgaacggggg gttcgtgcac acagcccagc ttggagcgaa

1921 cgacctacac cgaactgaga tacctacagc gtgagctatg agaaagcgcc acgcttcccg

1981 aagggagaaa ggcggacagg tatccggtaa gcggcagggt cggaacagga gagcgcacga

2041 gggagcttcc agggggaaac gcctggtatc tttatagtcc tgtcgggttt cgccacctct

2101 gacttgagcg tcgatttttg tgatgctcgt caggggggcg gagcctatgg aaaaacgcca

2161 gcaacgcggc ctttttacgg ttcctggcct tttgctggcc ttttgctcac atgttctttc

2221 ctgcgttatc ccctgattct gtggataacc gtattaccgc ctttgagtga gctgataccg

2281 ctcgccgcag ccgaacgacc gagcgcagcg agtcagtgag cgaggaagcg gaagagcgcc

2341 tgatgcggta ttttctcctt acgcatctgt gcggtatttc acaccgcata tggtgcactc

2401 tcagtacaat ctgctctgat gccgcatagt taagccagta tacactccgc tatcgctacg

2461 tgactgggtc atggctgcgc cccgacaccc gccaacaccc gctgacgcgc cctgacgggc

2521 ttgtctgctc ccggcatccg cttacagaca agctgtgacc gtctccggga gctgcatgtg

2581 tcagaggttt tcaccgtcat caccgaaacg cgcgaggcag ggtgccttga tgtgggcgcc

2641 ggcggtcgag tggcgacggc gcggcttgtc cgcgccctgg tagattgcct ggccgtaggc

2701 cagccatttt tgagcggcca gcggccgcga taggccgacg cgaagcggcg gggcgtaggg

2761 agcgcagcga ccgaagggta ggcgcttttt gcagctcttc ggctgtgcgc tggccagaca

2821 gttatgcaca ggccaggcgg gttttaagag ttttaataag ttttaaagag ttttaggcgg

2881 aaaaatcgcc ttttttctct tttatatcag tcacttacat gtgtgaccgg ttcccaatgt

2941 acggctttgg gttcccaatg tacgggttcc ggttcccaat gtacggcttt gggttcccaa

3001 tgtacgtgct atccacagga aagagtcctt ttcgaccttt ttcccctgct agggcaattt

3061 gccctagcat ctgctccgta cattaggaac cggcggatgc ttcgccctcg atcaggttgc

3121 ggtagcgcat gactaggatc gggccagcct gccccgcctc ctccttcaaa tcgtactccg

3181 gcaggtcatt tgacccgatc agcttgcgca cggtgaaaca gaacttcttg aactctccgg

3241 cgctgccact gcgttcgtag atcgtcttga acaaccatct ggcttctgcc ttgcctgcgg

3301 cgcggcgtgc caggcggtag agaaaacggc cgatgccggg atcgatcaaa aagtaatcgg

3361 ggtgaaccgt cagcacgtcc gggttcttgc cttctgtgat ctcgcggtac atccaatcag

3421 ctagctcgat ctcgatgtac tccggccgcc cggtttcgct ctttacgatc ttgtagcggc

3481 taatcaaggc ttcaccctcg gataccgtca ccaggcggcc gttcttggcc ttcttcgtac

3541 gctgcatggc aacgtgcgtg gtgtttaacc gaatgcaggt ttctaccagg tcgtctttct

3601 gctttccgcc atcggctcgc cggcagaact tgagtacgtc cgcaacgtgt ggacggaaca

3661 cgcggccggg cttgtctccc ttcccttccc ggtatcggtt catggattcg gttagatggg

3721 aaaccgccat cagtaccagg tcgtaatccc acacactggc catgccggcc ggccctgcgg

3781 aaacctctac gtgcccgtct ggaagctcgt agcggatcac ctcgccagct cgtcggtcac

3841 gcttcgacag acggaaaacg gccacgtcca tgatgctgcg actatcgcgg gtgcccacgt

3901 catagagcat cggaacgaaa aaatctggtt gctcgtcgcc cttgggcggc ttcctaatcg

3961 acggcgcacc ggctgccggc ggttgccggg attctttgcg gattcgatca gcggccgctt

4021 gccacgattc accggggcgt gcttctgcct cgatgcgttg ccgctgggcg gcctgcgcgg

4081 ccttcaactt ctccaccagg tcatcaccca gcgccgcgcc gatttgtacc gggccggatg

4141 gtttgcgacc gtcacgccga ttcctcgggc ttgggggttc cagtgccatt gcagggccgg

4201 cagacaaccc agccgcttac gcctggccaa ccgcccgttc ctccacacat ggggcattcc

4261 acggcgtcgg tgcctggttg ttcttgattt tccatgccgc ctcctttagc cgctaaaatt

4321 catctactca tttattcatt tgctcattta ctctggtagc tgcgcgatgt attcagatag

4381 cagctcggta atggtcttgc cttggcgtac cgcgtacatc ttcagcttgg tgtgatcctc

4441 cgccggcaac tgaaagttga cccgcttcat ggctggcgtg tctgccaggc tggccaacgt

4501 tgcagccttg ctgctgcgtg cgctcggacg gccggcactt agcgtgtttg tgcttttgct

4561 cattttctct ttacctcatt aactcaaatg agttttgatt taatttcagc ggccagcgcc

4621 tggacctcgc gggcagcgtc gccctcgggt tctgattcaa gaacggttgt gccggcggcg

4681 gcagtgcctg ggtagctcac gcgctgcgtg atacgggact caagaatggg cagctcgtac

4741 ccggccagcg cctcggcaac ctcaccgccg atgcgcgtgc ctttgatcgc ccgcgacacg

4801 acaaaggccg cttgtagcct tccatccgtg acctcaatgc gctgcttaac cagctccacc

4861 aggtcggcgg tggcccatat gtcgtaaggg cttggctgca ccggaatcag cacgaagtcg

4921 gctgccttga tcgcggacac agccaagtcc gccgcctggg gcgctccgtc gatcactacg

4981 aagtcgcgcc ggccgatggc cttcacgtcg cggtcaatcg tcgggcggtc gatgccgaca

5041 acggttagcg gttgatcttc ccgcacggcc gcccaatcgc gggcactgcc ctggggatcg

5101 gaatcgacta acagaacatc ggccccggcg agttgcaggg cgcgggctag atgggttgcg

5161 atggtcgtct tgcctgaccc gcctttctgg ttaagtacag cgataacctt catgcgttcc

5221 ccttgcgtat ttgtttattt actcatcgca tcatatacgc agcgaccgca tgacgcaagc

5281 tgttttactc aaatacacat caccttttta gacggcggcg ctcggtttct tcagcggcca

5341 agctggccgg ccaggccgcc agcttggcat cagacaaacc ggccaggatt tcatgcagcc

5401 gcacggttga gacgtgcgcg ggcggctcga acacgtaccc ggccgcgatc atctccgcct

5461 cgatctcttc ggtaatgaaa aacggttcgt cctggccgtc ctggtgcggt ttcatgcttg

5521 ttcctcttgg cgttcattct cggcggccgc cagggcgtcg gcctcggtca atgcgtcctc

5581 acggaaggca ccgcgccgcc tggcctcggt gggcgtcact tcctcgctgc gctcaagtgc

5641 gcggtacagg gtcgagcgat gcacgccaag cagtgcagcc gcctctttca cggtgcggcc

5701 ttcctggtcg atcagctcgc gggcgtgcgc gatctgtgcc ggggtgaggg tagggcgggg

5761 gccaaacttc acgcctcggg ccttggcggc ctcgcgcccg ctccgggtgc ggtcgatgat

5821 tagggaacgc tcgaactcgg caatgccggc gaacacggtc aacaccatgc ggccggccgg

5881 cgtggtggtg tcggcccacg gctctgccag gctacgcagg cccgcgccgg cctcctggat

5941 gcgctcggca atgtccagta ggtcgcgggt gctgcgggcc aggcggtcta gcctggtcac

6001 tgtcacaacg tcgccagggc gtaggtggtc aagcatcctg gccagctccg ggcggtcgcg

6061 cctggtgccg gtgatcttct cggaaaacag cttggtgcag ccggccgcgt gcagttcggc

6121 ccgttggttg gtcaagtcct ggtcgtcggt gctgacgcgg gcatagccca gcaggccagc

6181 ggcggcgctc ttgttcatgg cgtaatgtct ccggttctag tcgcaagtat tctactttat

6241 gcgactaaaa cacgcgacaa gaaaacgcca ggaaaagggc agggcggcag cctgtcgcgt

6301 aacttaggac ttgtgcgaca tgtcgttttc agaagacggc tgcactgaac gtcagaagcc

6361 gactgcacta tagcagcgga ggggttggat caaagtactt tgatcccgag gggaaccctg

6421 tggttggcat gcacatacaa atggacgaac ggataaacct tttcacgccc ttttaaatat

6481 ccgttattct aataaacgct cttttctctt aggtttaccc gccaatatat cctgtcaaac

6541 actgatagtt taaactgaag gcgggaaacg acaatctgat ccaagctcaa gctgctctag

6601 cattcgccat tcaggctgcg caactgttgg gaagggcgat cggtgcgggc ctcttcgcta

6661 ttacgccagc tggcgaaagg gggatgtgct gcaaggcgat taagttgggt aacgccaggg

6721 ttttcccagt cacgacgttg taaaacgacg gccagtgcca agcttcattc ggagtttttg

6781 tatcttgttt catagtttgt cccaggatta gaatgattag gcatcgaacc ttcaagaatt

6841 tgattgaata aaacatcttc attcttaaga tatgaagata atcttcaaaa ggcccctggg

6901 aatctgaaag aagagaagca ggcccattta tatgggaaag aacaatagta tttcttatat

6961 aggcccattt aagttgaaaa caatcttcaa aagtcccaca tcgcttagat aagaaaacga

7021 agctgagttt atatacagct agagtcgaag tagtgattgg agaccgaggt ctctgtttta

7081 gagctagaaa tagcaagtta aaataaggct agtccgttat caacttgaaa aagtggcacc

7141 gagtcggtgc ttttttgttt tagagctaga aatagcaagt taaaataagg ctagtccgta

7201 ggcgcgccaa ttctgcagac aaatggcccc gggatatttc acaaattgaa catagactac

7261 agaattttag aaaacaaact ttctctctct tatctcacct ttatctttta gagagaaaaa

7321 gttcgatttc cggttgaccg gaatgtatct ttgttttttt tgttttgtaa catatttcgt

7381 tttccgattt agatcggatc tccttttccg ttttgtcgga ccttcttccg gtttatccgg

7441 atctaataat atccatctta gacttagcta agtttggatc tgttttttgg ttagctcttg

7501 tcaatcgcct catcatcagc aagaaggtga aatttttgac aaataaatct tagaatcatg

7561 tagtgtcttt ggaccttggg aatgatagaa acgatttgtt atagctactc tatgtatcag

7621 accctgacca agatccaaca atctcatagg ttttgtgcat atgaaacctt cgactaacga

7681 gaagtggtct tttaatgaga gagatatcta aaatgttatc ttaaaagccc actcaaatct

7741 caaggcataa ggtagaaatg caaatttgga aagtgggctg ggccttttgt ggtaaaggcc

7801 tgtaacctag cccaatatta gcaaaaccct agacgcgtac attgacatat ataaacccgc

7861 ctcctccttg tttagggttt ctacgtgaga gagacgaaac acaaaccatg gactataagg

7921 accacgacgg agactacaag gatcatgata ttgattacaa agacgatgac gataagatgg

7981 ccccaaagaa gaagcggaag gtcggtatcc acggagtccc agcagccgac aagaagtaca

8041 gcatcggcct ggacatcggc accaactctg tgggctgggc cgtgatcacc gacgagtaca

8101 aggtgcccag caagaaattc aaggtgctgg gcaacaccga ccggcacagc atcaagaaga

8161 acctgatcgg agccctgctg ttcgacagcg gcgaaacagc cgaggccacc cggctgaaga

8221 gaaccgccag aagaagatac accagacgga agaaccggat ctgctatctg caagagatct

8281 tcagcaacga gatggccaag gtggacgaca gcttcttcca cagactggaa gagtccttcc

8341 tggtggaaga ggataagaag cacgagcggc accccatctt cggcaacatc gtggacgagg

8401 tggcctacca cgagaagtac cccaccatct accacctgag aaagaaactg gtggacagca

8461 ccgacaaggc cgacctgcgg ctgatctatc tggccctggc ccacatgatc aagttccggg

8521 gccacttcct gatcgagggc gacctgaacc ccgacaacag cgacgtggac aagctgttca

8581 tccagctggt gcagacctac aaccagctgt tcgaggaaaa ccccatcaac gccagcggcg

8641 tggacgccaa ggccatcctg tctgccagac tgagcaagag cagacggctg gaaaatctga

8701 tcgcccagct gcccggcgag aagaagaatg gcctgttcgg caacctgatt gccctgagcc

8761 tgggcctgac ccccaacttc aagagcaact tcgacctggc cgaggatgcc aaactgcagc

8821 tgagcaagga cacctacgac gacgacctgg acaacctgct ggcccagatc ggcgaccagt

8881 acgccgacct gtttctggcc gccaagaacc tgtccgacgc catcctgctg agcgacatcc

8941 tgagagtgaa caccgagatc accaaggccc ccctgagcgc ctctatgatc aagagatacg

9001 acgagcacca ccaggacctg accctgctga aagctctcgt gcggcagcag ctgcctgaga

9061 agtacaaaga gattttcttc gaccagagca agaacggcta cgccggctac attgacggcg

9121 gagccagcca ggaagagttc tacaagttca tcaagcccat cctggaaaag atggacggca

9181 ccgaggaact gctcgtgaag ctgaacagag aggacctgct gcggaagcag cggaccttcg

9241 acaacggcag catcccccac cagatccacc tgggagagct gcacgccatt ctgcggcggc

9301 aggaagattt ttacccattc ctgaaggaca accgggaaaa gatcgagaag atcctgacct

9361 tccgcatccc ctactacgtg ggccctctgg ccaggggaaa cagcagattc gcctggatga

9421 ccagaaagag cgaggaaacc atcaccccct ggaacttcga ggaagtggtg gacaagggcg

9481 cttccgccca gagcttcatc gagcggatga ccaacttcga taagaacctg cccaacgaga

9541 aggtgctgcc caagcacagc ctgctgtacg agtacttcac cgtgtataac gagctgacca

9601 aagtgaaata cgtgaccgag ggaatgagaa agcccgcctt cctgagcggc gagcagaaaa

9661 aggccatcgt ggacctgctg ttcaagacca accggaaagt gaccgtgaag cagctgaaag

9721 aggactactt caagaaaatc gagtgcttcg actccgtgga aatctccggc gtggaagatc

9781 ggttcaacgc ctccctgggc acataccacg atctgctgaa aattatcaag gacaaggact

9841 tcctggacaa tgaggaaaac gaggacattc tggaagatat cgtgctgacc ctgacactgt

9901 ttgaggacag agagatgatc gaggaacggc tgaaaaccta tgcccacctg ttcgacgaca

9961 aagtgatgaa gcagctgaag cggcggagat acaccggctg gggcaggctg agccggaagc

10021 tgatcaacgg catccgggac aagcagtccg gcaagacaat cctggatttc ctgaagtccg

10081 acggcttcgc caacagaaac ttcatgcagc tgatccacga cgacagcctg acctttaaag

10141 aggacatcca gaaagcccag gtgtccggcc agggcgatag cctgcacgag cacattgcca

10201 atctggccgg cagccccgcc attaagaagg gcatcctgca gacagtgaag gtggtggacg

10261 agctcgtgaa agtgatgggc cggcacaagc ccgagaacat cgtgatcgaa atggccagag

10321 agaaccagac cacccagaag ggacagaaga acagccgcga gagaatgaag cggatcgaag

10381 agggcatcaa agagctgggc agccagatcc tgaaagaaca ccccgtggaa aacacccagc

10441 tgcagaacga gaagctgtac ctgtactacc tgcagaatgg gcgggatatg tacgtggacc

10501 aggaactgga catcaaccgg ctgtccgact acgatgtgga ccatatcgtg cctcagagct

10561 ttctgaagga cgactccatc gacaacaagg tgctgaccag aagcgacaag aaccggggca

10621 agagcgacaa cgtgccctcc gaagaggtcg tgaagaagat gaagaactac tggcggcagc

10681 tgctgaacgc caagctgatt acccagagaa agttcgacaa tctgaccaag gccgagagag

10741 gcggcctgag cgaactggat aaggccggct tcatcaagag acagctggtg gaaacccggc

10801 agatcacaaa gcacgtggca cagatcctgg actcccggat gaacactaag tacgacgaga

10861 atgacaagct gatccgggaa gtgaaagtga tcaccctgaa gtccaagctg gtgtccgatt

10921 tccggaagga tttccagttt tacaaagtgc gcgagatcaa caactaccac cacgcccacg

10981 acgcctacct gaacgccgtc gtgggaaccg ccctgatcaa aaagtaccct aagctggaaa

11041 gcgagttcgt gtacggcgac tacaaggtgt acgacgtgcg gaagatgatc gccaagagcg

11101 agcaggaaat cggcaaggct accgccaagt acttcttcta cagcaacatc atgaactttt

11161 tcaagaccga gattaccctg gccaacggcg agatccggaa gcggcctctg atcgagacaa

11221 acggcgaaac cggggagatc gtgtgggata agggccggga ttttgccacc gtgcggaaag

11281 tgctgagcat gccccaagtg aatatcgtga aaaagaccga ggtgcagaca ggcggcttca

11341 gcaaagagtc tatcctgccc aagaggaaca gcgataagct gatcgccaga aagaaggact

11401 gggaccctaa gaagtacggc ggcttcgaca gccccaccgt ggcctattct gtgctggtgg

11461 tggccaaagt ggaaaagggc aagtccaaga aactgaagag tgtgaaagag ctgctgggga

11521 tcaccatcat ggaaagaagc agcttcgaga agaatcccat cgactttctg gaagccaagg

11581 gctacaaaga agtgaaaaag gacctgatca tcaagctgcc taagtactcc ctgttcgagc

11641 tggaaaacgg ccggaagaga atgctggcct ctgccggcga actgcagaag ggaaacgaac

11701 tggccctgcc ctccaaatat gtgaacttcc tgtacctggc cagccactat gagaagctga

11761 agggctcccc cgaggataat gagcagaaac agctgtttgt ggaacagcac aagcactacc

11821 tggacgagat catcgagcag atcagcgagt tctccaagag agtgatcctg gccgacgcta

11881 atctggacaa agtgctgtcc gcctacaaca agcaccggga taagcccatc agagagcagg

11941 ccgagaatat catccacctg tttaccctga ccaatctggg agcccctgcc gccttcaagt

12001 actttgacac caccatcgac cggaagaggt acaccagcac caaagaggtg ctggacgcca

12061 ccctgatcca ccagagcatc accggcctgt acgagacacg gatcgacctg tctcagctgg

12121 gaggcgacaa aaggccggcg gccacgaaaa aggccggcca ggcaaaaaag aaaaagtaag

12181 gatccagaga ctcttatcaa gaatcccatc tcttgcttgc ttttttttgt tgcttccctt

12241 tgatagggtt tgtttttctt gtttcagtga ctttctatgt taaaagataa tgtcagtaaa

12301 aggatttggt tttctattat tctgaatcga ttacggaaga ttcttgctta attccaatct

12361 atacaagtat cgtgaaataa tgaccgttta tgtggtaccg agctcgaatt cgtaatcatg

12421 gtcatagctg tttcctgtgt gaaattgtta tccgctcaca attccacaca acatacgagc

12481 cggaagcata aagtgtaaag cctggggtgc ctaatgagtg agctaactca cattaattgc

12541 gttgcgctca ctgcccgctt tccagtcggg aaacctgtcg tgccagctgc attaatgaat

12601 cggccaacgc gcggggagag gcggtttgcg tattggctag agcagcttgc caacatggtg

12661 gagcacgaca ctctcgtcta ctccaagaat atcaaagata cagtctcaga agaccaaagg

12721 gctattgaga cttttcaaca aagggtaata tcgggaaacc tcctcggatt ccattgccca

12781 gctatctgtc acttcatcaa aaggacagta gaaaaggaag gtggcaccta caaatgccat

12841 cattgcgata aaggaaaggc tatcgttcaa gatgcctctg ccgacagtgg tcccaaagat

12901 ggacccccac ccacgaggag catcgtggaa aaagaagacg ttccaaccac gtcttcaaag

12961 caagtggatt gatgtgataa catggtggag cacgacactc tcgtctactc caagaatatc

13021 aaagatacag tctcagaaga ccaaagggct attgagactt ttcaacaaag ggtaatatcg

13081 ggaaacctcc tcggattcca ttgcccagct atctgtcact tcatcaaaag gacagtagaa

13141 aaggaaggtg gcacctacaa atgccatcat tgcgataaag gaaaggctat cgttcaagat

13201 gcctctgccg acagtggtcc caaagatgga cccccaccca cgaggagcat cgtggaaaaa

13261 gaagacgttc caaccacgtc ttcaaagcaa gtggattgat gtgatatctc cactgacgta

13321 agggatgacg cacaatccca ctatccttcg caagaccttc ctctatataa ggaagttcat

13381 ttcatttgga gaggacacgc tgaaatcacc agtctctctc tacaaatcta tctctctcga

13441 gatggtgagc aagggcgagg agctgttcac cggggtggtg cccatcctgg tcgagctgga

13501 cggcgacgta aacggccaca agttcagcgt gtccggcgag ggcgagggcg atgccaccta

13561 cggcaagctg accctgaagt tcatctgcac caccggcaag ctgcccgtgc cctggcccac

13621 cctcgtgacc accctgacct acggcgtgca gtgcttcagc cgctaccccg accacatgaa

13681 gcagcacgac ttcttcaagt ccgccatgcc cgaaggctac gtccaggagc gcaccatctt

13741 cttcaaggac gacggcaact acaagacccg cgccgaggtg aagttcgagg gcgacaccct

13801 ggtgaaccgc atcgagctga agggcatcga cttcaaggag gacggcaaca tcctggggca

13861 caagctggag tacaactaca acagccacaa cgtctatatc atggccgaca agcagaagaa

13921 cggcatcaag gtgaacttca agatccgcca caacatcgag gacggcagcg tgcagctcgc

13981 cgaccactac cagcagaaca cccccatcgg cgacggcccc gtgctgctgc ccgacaacca

14041 ctacctgagc acccagtccg ccctgagcaa agaccccaac gagaagcgcg atcacatggt

14101 cctgctggag ttcgtgaccg ccgccgggat cactctcggc atggacgagc tgtacaagta

14161 actcgagttt ctccataata atgtg

//

**1-step Cas9 AtU6 mCherry vector Genbank sequence, including the metadata and annotations.**

LOCUS 1_step_Cas9_AtU6 14176 bp DNA circular UNA 03-MAR-2025

DEFINITION Gibson Assembly of PSC1-BplU3-4 - BlpR=PCR 产物 into Digestion of

pCAMBIA1300-psgR (modified)= Xhol.

ACCESSION urn.local...cm-ivpv22s

VERSION urn.local...cm-ivpv22s

KEYWORDS .

SOURCE

ORGANISM

.

FEATURES Location/Qualifiers

misc_feature complement(<14158..167)

/label="Misc_Feature_6"

misc_feature complement(234..259)

/label="Misc_Feature_5"

CDS 684..1478

/label="CDS_2"

rep_origin 1769..2049

/label="Rep_Origin_2"

misc_feature 2189..2449

/label="Misc_Feature_4"

rep_origin 2859..3859

/label="Rep_Origin_1"

misc_difference 3026

/label="T to A"

misc_feature 4452..5452

/label="Misc_Feature_3"

misc_feature complement(6493..6518)

/label="Misc_Feature_2"

misc_feature complement(6738..>6760)

/label="Misc_Feature_1"

promoter 6766..7058

/label="AtU6"

ncRNA 7059..7156

/label="ncRNA"

promoter 7228..7907

/label="pAtUBQ1"

misc_feature 7908..8027

/note="longNLS"

/label="longNLS"

misc_feature 8028..12128

/note="hSpCsn1"

/modified_by="User"

/label="hSpCsn1"

misc_feature 8052..8054

/note="D10"

/label="D10"

misc_feature 12129..12176

/note="NLS"

/label="NLS"

terminator 12186..12393

/label="tUBQ1"

origin 12406^12407

/label="origin"

misc_feature complement(12453..12507)

/label="Promoter_P_2"

/note="Geneious type: promoter prokaryotic"

misc_feature 12655..13435

/label="Promoter_P_1"

/note="Geneious type: promoter prokaryotic"

ligation 13418..13436

/label="Ligation"

primer_bind 13418..13441

/Sequence="CTCTACAAATCTATCTCTCTCGAG"

/Hairpin_Tm="38.7"

/Tm="55.5"

/Self_Dimer_Tm="5.9"

/%GC="41.7"

/modified_by="User"

/label="Binding Region"

primer_bind 13442..13459

/Sequence="ATGGTGAGCAAGGGCGAG"

/Hairpin_Tm="47.5"

/Tm="59.7"

/Self_Dimer_Tm="None"

/%GC="61.1"

/created_by="primer3"

/modified_by="User"

/label="MCHERRY-F"

primer_bind complement(14132..14152)

/Sequence="CTACTTGTACAGCTCGTCCAT"

/Hairpin_Tm="None"

/Tm="57.2"

/Self_Dimer_Tm="None"

/%GC="47.6"

/created_by="primer3"

/modified_by="User"

/label="MCHERRY-R"

misc_feature <14153..14176

/label="PSC1-BplU3-4 - BlpR=PCR 产物"

/note="Geneious type: Inserted sequence"

primer_bind 14153..14176

/created_by="primer3"

/annotation_group="441838253606200dd92c503-e200-44ee-9a5c-

4f9bc535cbd5"

/%GC="37.5"

/Tm="55.5"

/Hairpin_Tm="None"

/Self_Dimer_Tm="3.6"

/Sequence="CTCGAGTTTCTCCATAATAATGTG"

/label="Binding Region"

ligation 14158..14176

/label="Ligation"

ORIGIN

1 tgagtagttc ccagataagg gaattagggt tcctataggg tttcgctcat gtgttgagca

61 tataagaaac ccttagtatg tatttgtatt tgtaaaatac ttctatcaat aaaatttcta

121 attcctaaaa ccaaaatcca gtactaaaat ccagatcccc cgaattaatt cggcgttaat

181 tcagtacatt aaaaacgtcc gcaatgtgtt attaagttgt ctaagcgtca atttgtttac

241 accacaatat atcctgccac cagccagcca acagctcccc gaccggcagc tcggcacaaa

301 atcaccactc gatacaggca gcccatcagt ccgggacggc gtcagcggga gagccgttgt

361 aaggcggcag actttgctca tgttaccgat gctattcgga agaacggcaa ctaagctgcc

421 gggtttgaaa cacggatgat ctcgcggagg gtagcatgtt gattgtaacg atgacagagc

481 gttgctgcct gtgatcaccg cggtttcaaa atcggctccg tcgatactat gttatacgcc

541 aactttgaaa acaactttga aaaagctgtt ttctggtatt taaggtttta gaatgcaagg

601 aacagtgaat tggagttcgt cttgttataa ttagcttctt ggggtatctt taaatactgt

661 agaaaagagg aaggaaataa taaatggcta aaatgagaat atcaccggaa ttgaaaaaac

721 tgatcgaaaa ataccgctgc gtaaaagata cggaaggaat gtctcctgct aaggtatata

781 agctggtggg agaaaatgaa aacctatatt taaaaatgac ggacagccgg tataaaggga

841 ccacctatga tgtggaacgg gaaaaggaca tgatgctatg gctggaagga aagctgcctg

901 ttccaaaggt cctgcacttt gaacggcatg atggctggag caatctgctc atgagtgagg

961 ccgatggcgt cctttgctcg gaagagtatg aagatgaaca aagccctgaa aagattatcg

1021 agctgtatgc ggagtgcatc aggctctttc actccatcga catatcggat tgtccctata

1081 cgaatagctt agacagccgc ttagccgaat tggattactt actgaataac gatctggccg

1141 atgtggattg cgaaaactgg gaagaagaca ctccatttaa agatccgcgc gagctgtatg

1201 attttttaaa gacggaaaag cccgaagagg aacttgtctt ttcccacggc gacctgggag

1261 acagcaacat ctttgtgaaa gatggcaaag taagtggctt tattgatctt gggagaagcg

1321 gcagggcgga caagtggtat gacattgcct tctgcgtccg gtcgatcagg gaggatatcg

1381 gggaagaaca gtatgtcgag ctattttttg acttactggg gatcaagcct gattgggaga

1441 aaataaaata ttatatttta ctggatgaat tgttttagta cctagaatgc atgaccaaaa

1501 tcccttaacg tgagttttcg ttccactgag cgtcagaccc cgtagaaaag atcaaaggat

1561 cttcttgaga tccttttttt ctgcgcgtaa tctgctgctt gcaaacaaaa aaaccaccgc

1621 taccagcggt ggtttgtttg ccggatcaag agctaccaac tctttttccg aaggtaactg

1681 gcttcagcag agcgcagata ccaaatactg tccttctagt gtagccgtag ttaggccacc

1741 acttcaagaa ctctgtagca ccgcctacat acctcgctct gctaatcctg ttaccagtgg

1801 ctgctgccag tggcgataag tcgtgtctta ccgggttgga ctcaagacga tagttaccgg

1861 ataaggcgca gcggtcgggc tgaacggggg gttcgtgcac acagcccagc ttggagcgaa

1921 cgacctacac cgaactgaga tacctacagc gtgagctatg agaaagcgcc acgcttcccg

1981 aagggagaaa ggcggacagg tatccggtaa gcggcagggt cggaacagga gagcgcacga

2041 gggagcttcc agggggaaac gcctggtatc tttatagtcc tgtcgggttt cgccacctct

2101 gacttgagcg tcgatttttg tgatgctcgt caggggggcg gagcctatgg aaaaacgcca

2161 gcaacgcggc ctttttacgg ttcctggcct tttgctggcc ttttgctcac atgttctttc

2221 ctgcgttatc ccctgattct gtggataacc gtattaccgc ctttgagtga gctgataccg

2281 ctcgccgcag ccgaacgacc gagcgcagcg agtcagtgag cgaggaagcg gaagagcgcc

2341 tgatgcggta ttttctcctt acgcatctgt gcggtatttc acaccgcata tggtgcactc

2401 tcagtacaat ctgctctgat gccgcatagt taagccagta tacactccgc tatcgctacg

2461 tgactgggtc atggctgcgc cccgacaccc gccaacaccc gctgacgcgc cctgacgggc

2521 ttgtctgctc ccggcatccg cttacagaca agctgtgacc gtctccggga gctgcatgtg

2581 tcagaggttt tcaccgtcat caccgaaacg cgcgaggcag ggtgccttga tgtgggcgcc

2641 ggcggtcgag tggcgacggc gcggcttgtc cgcgccctgg tagattgcct ggccgtaggc

2701 cagccatttt tgagcggcca gcggccgcga taggccgacg cgaagcggcg gggcgtaggg

2761 agcgcagcga ccgaagggta ggcgcttttt gcagctcttc ggctgtgcgc tggccagaca

2821 gttatgcaca ggccaggcgg gttttaagag ttttaataag ttttaaagag ttttaggcgg

2881 aaaaatcgcc ttttttctct tttatatcag tcacttacat gtgtgaccgg ttcccaatgt

2941 acggctttgg gttcccaatg tacgggttcc ggttcccaat gtacggcttt gggttcccaa

3001 tgtacgtgct atccacagga aagagtcctt ttcgaccttt ttcccctgct agggcaattt

3061 gccctagcat ctgctccgta cattaggaac cggcggatgc ttcgccctcg atcaggttgc

3121 ggtagcgcat gactaggatc gggccagcct gccccgcctc ctccttcaaa tcgtactccg

3181 gcaggtcatt tgacccgatc agcttgcgca cggtgaaaca gaacttcttg aactctccgg

3241 cgctgccact gcgttcgtag atcgtcttga acaaccatct ggcttctgcc ttgcctgcgg

3301 cgcggcgtgc caggcggtag agaaaacggc cgatgccggg atcgatcaaa aagtaatcgg

3361 ggtgaaccgt cagcacgtcc gggttcttgc cttctgtgat ctcgcggtac atccaatcag

3421 ctagctcgat ctcgatgtac tccggccgcc cggtttcgct ctttacgatc ttgtagcggc

3481 taatcaaggc ttcaccctcg gataccgtca ccaggcggcc gttcttggcc ttcttcgtac

3541 gctgcatggc aacgtgcgtg gtgtttaacc gaatgcaggt ttctaccagg tcgtctttct

3601 gctttccgcc atcggctcgc cggcagaact tgagtacgtc cgcaacgtgt ggacggaaca

3661 cgcggccggg cttgtctccc ttcccttccc ggtatcggtt catggattcg gttagatggg

3721 aaaccgccat cagtaccagg tcgtaatccc acacactggc catgccggcc ggccctgcgg

3781 aaacctctac gtgcccgtct ggaagctcgt agcggatcac ctcgccagct cgtcggtcac

3841 gcttcgacag acggaaaacg gccacgtcca tgatgctgcg actatcgcgg gtgcccacgt

3901 catagagcat cggaacgaaa aaatctggtt gctcgtcgcc cttgggcggc ttcctaatcg

3961 acggcgcacc ggctgccggc ggttgccggg attctttgcg gattcgatca gcggccgctt

4021 gccacgattc accggggcgt gcttctgcct cgatgcgttg ccgctgggcg gcctgcgcgg

4081 ccttcaactt ctccaccagg tcatcaccca gcgccgcgcc gatttgtacc gggccggatg

4141 gtttgcgacc gtcacgccga ttcctcgggc ttgggggttc cagtgccatt gcagggccgg

4201 cagacaaccc agccgcttac gcctggccaa ccgcccgttc ctccacacat ggggcattcc

4261 acggcgtcgg tgcctggttg ttcttgattt tccatgccgc ctcctttagc cgctaaaatt

4321 catctactca tttattcatt tgctcattta ctctggtagc tgcgcgatgt attcagatag

4381 cagctcggta atggtcttgc cttggcgtac cgcgtacatc ttcagcttgg tgtgatcctc

4441 cgccggcaac tgaaagttga cccgcttcat ggctggcgtg tctgccaggc tggccaacgt

4501 tgcagccttg ctgctgcgtg cgctcggacg gccggcactt agcgtgtttg tgcttttgct

4561 cattttctct ttacctcatt aactcaaatg agttttgatt taatttcagc ggccagcgcc

4621 tggacctcgc gggcagcgtc gccctcgggt tctgattcaa gaacggttgt gccggcggcg

4681 gcagtgcctg ggtagctcac gcgctgcgtg atacgggact caagaatggg cagctcgtac

4741 ccggccagcg cctcggcaac ctcaccgccg atgcgcgtgc ctttgatcgc ccgcgacacg

4801 acaaaggccg cttgtagcct tccatccgtg acctcaatgc gctgcttaac cagctccacc

4861 aggtcggcgg tggcccatat gtcgtaaggg cttggctgca ccggaatcag cacgaagtcg

4921 gctgccttga tcgcggacac agccaagtcc gccgcctggg gcgctccgtc gatcactacg

4981 aagtcgcgcc ggccgatggc cttcacgtcg cggtcaatcg tcgggcggtc gatgccgaca

5041 acggttagcg gttgatcttc ccgcacggcc gcccaatcgc gggcactgcc ctggggatcg

5101 gaatcgacta acagaacatc ggccccggcg agttgcaggg cgcgggctag atgggttgcg

5161 atggtcgtct tgcctgaccc gcctttctgg ttaagtacag cgataacctt catgcgttcc

5221 ccttgcgtat ttgtttattt actcatcgca tcatatacgc agcgaccgca tgacgcaagc

5281 tgttttactc aaatacacat caccttttta gacggcggcg ctcggtttct tcagcggcca

5341 agctggccgg ccaggccgcc agcttggcat cagacaaacc ggccaggatt tcatgcagcc

5401 gcacggttga gacgtgcgcg ggcggctcga acacgtaccc ggccgcgatc atctccgcct

5461 cgatctcttc ggtaatgaaa aacggttcgt cctggccgtc ctggtgcggt ttcatgcttg

5521 ttcctcttgg cgttcattct cggcggccgc cagggcgtcg gcctcggtca atgcgtcctc

5581 acggaaggca ccgcgccgcc tggcctcggt gggcgtcact tcctcgctgc gctcaagtgc

5641 gcggtacagg gtcgagcgat gcacgccaag cagtgcagcc gcctctttca cggtgcggcc

5701 ttcctggtcg atcagctcgc gggcgtgcgc gatctgtgcc ggggtgaggg tagggcgggg

5761 gccaaacttc acgcctcggg ccttggcggc ctcgcgcccg ctccgggtgc ggtcgatgat

5821 tagggaacgc tcgaactcgg caatgccggc gaacacggtc aacaccatgc ggccggccgg

5881 cgtggtggtg tcggcccacg gctctgccag gctacgcagg cccgcgccgg cctcctggat

5941 gcgctcggca atgtccagta ggtcgcgggt gctgcgggcc aggcggtcta gcctggtcac

6001 tgtcacaacg tcgccagggc gtaggtggtc aagcatcctg gccagctccg ggcggtcgcg

6061 cctggtgccg gtgatcttct cggaaaacag cttggtgcag ccggccgcgt gcagttcggc

6121 ccgttggttg gtcaagtcct ggtcgtcggt gctgacgcgg gcatagccca gcaggccagc

6181 ggcggcgctc ttgttcatgg cgtaatgtct ccggttctag tcgcaagtat tctactttat

6241 gcgactaaaa cacgcgacaa gaaaacgcca ggaaaagggc agggcggcag cctgtcgcgt

6301 aacttaggac ttgtgcgaca tgtcgttttc agaagacggc tgcactgaac gtcagaagcc

6361 gactgcacta tagcagcgga ggggttggat caaagtactt tgatcccgag gggaaccctg

6421 tggttggcat gcacatacaa atggacgaac ggataaacct tttcacgccc ttttaaatat

6481 ccgttattct aataaacgct cttttctctt aggtttaccc gccaatatat cctgtcaaac

6541 actgatagtt taaactgaag gcgggaaacg acaatctgat ccaagctcaa gctgctctag

6601 cattcgccat tcaggctgcg caactgttgg gaagggcgat cggtgcgggc ctcttcgcta

6661 ttacgccagc tggcgaaagg gggatgtgct gcaaggcgat taagttgggt aacgccaggg

6721 ttttcccagt cacgacgttg taaaacgacg gccagtgcca agcttcattc ggagtttttg

6781 tatcttgttt catagtttgt cccaggatta gaatgattag gcatcgaacc ttcaagaatt

6841 tgattgaata aaacatcttc attcttaaga tatgaagata atcttcaaaa ggcccctggg

6901 aatctgaaag aagagaagca ggcccattta tatgggaaag aacaatagta tttcttatat

6961 aggcccattt aagttgaaaa caatcttcaa aagtcccaca tcgcttagat aagaaaacga

7021 agctgagttt atatacagct agagtcgaag tagtgattgg agaccgaggt ctctgtttta

7081 gagctagaaa tagcaagtta aaataaggct agtccgttat caacttgaaa aagtggcacc

7141 gagtcggtgc ttttttgttt tagagctaga aatagcaagt taaaataagg ctagtccgta

7201 ggcgcgccaa ttctgcagac aaatggcccc gggatatttc acaaattgaa catagactac

7261 agaattttag aaaacaaact ttctctctct tatctcacct ttatctttta gagagaaaaa

7321 gttcgatttc cggttgaccg gaatgtatct ttgttttttt tgttttgtaa catatttcgt

7381 tttccgattt agatcggatc tccttttccg ttttgtcgga ccttcttccg gtttatccgg

7441 atctaataat atccatctta gacttagcta agtttggatc tgttttttgg ttagctcttg

7501 tcaatcgcct catcatcagc aagaaggtga aatttttgac aaataaatct tagaatcatg

7561 tagtgtcttt ggaccttggg aatgatagaa acgatttgtt atagctactc tatgtatcag

7621 accctgacca agatccaaca atctcatagg ttttgtgcat atgaaacctt cgactaacga

7681 gaagtggtct tttaatgaga gagatatcta aaatgttatc ttaaaagccc actcaaatct

7741 caaggcataa ggtagaaatg caaatttgga aagtgggctg ggccttttgt ggtaaaggcc

7801 tgtaacctag cccaatatta gcaaaaccct agacgcgtac attgacatat ataaacccgc

7861 ctcctccttg tttagggttt ctacgtgaga gagacgaaac acaaaccatg gactataagg

7921 accacgacgg agactacaag gatcatgata ttgattacaa agacgatgac gataagatgg

7981 ccccaaagaa gaagcggaag gtcggtatcc acggagtccc agcagccgac aagaagtaca

8041 gcatcggcct ggacatcggc accaactctg tgggctgggc cgtgatcacc gacgagtaca

8101 aggtgcccag caagaaattc aaggtgctgg gcaacaccga ccggcacagc atcaagaaga

8161 acctgatcgg agccctgctg ttcgacagcg gcgaaacagc cgaggccacc cggctgaaga

8221 gaaccgccag aagaagatac accagacgga agaaccggat ctgctatctg caagagatct

8281 tcagcaacga gatggccaag gtggacgaca gcttcttcca cagactggaa gagtccttcc

8341 tggtggaaga ggataagaag cacgagcggc accccatctt cggcaacatc gtggacgagg

8401 tggcctacca cgagaagtac cccaccatct accacctgag aaagaaactg gtggacagca

8461 ccgacaaggc cgacctgcgg ctgatctatc tggccctggc ccacatgatc aagttccggg

8521 gccacttcct gatcgagggc gacctgaacc ccgacaacag cgacgtggac aagctgttca

8581 tccagctggt gcagacctac aaccagctgt tcgaggaaaa ccccatcaac gccagcggcg

8641 tggacgccaa ggccatcctg tctgccagac tgagcaagag cagacggctg gaaaatctga

8701 tcgcccagct gcccggcgag aagaagaatg gcctgttcgg caacctgatt gccctgagcc

8761 tgggcctgac ccccaacttc aagagcaact tcgacctggc cgaggatgcc aaactgcagc

8821 tgagcaagga cacctacgac gacgacctgg acaacctgct ggcccagatc ggcgaccagt

8881 acgccgacct gtttctggcc gccaagaacc tgtccgacgc catcctgctg agcgacatcc

8941 tgagagtgaa caccgagatc accaaggccc ccctgagcgc ctctatgatc aagagatacg

9001 acgagcacca ccaggacctg accctgctga aagctctcgt gcggcagcag ctgcctgaga

9061 agtacaaaga gattttcttc gaccagagca agaacggcta cgccggctac attgacggcg

9121 gagccagcca ggaagagttc tacaagttca tcaagcccat cctggaaaag atggacggca

9181 ccgaggaact gctcgtgaag ctgaacagag aggacctgct gcggaagcag cggaccttcg

9241 acaacggcag catcccccac cagatccacc tgggagagct gcacgccatt ctgcggcggc

9301 aggaagattt ttacccattc ctgaaggaca accgggaaaa gatcgagaag atcctgacct

9361 tccgcatccc ctactacgtg ggccctctgg ccaggggaaa cagcagattc gcctggatga

9421 ccagaaagag cgaggaaacc atcaccccct ggaacttcga ggaagtggtg gacaagggcg

9481 cttccgccca gagcttcatc gagcggatga ccaacttcga taagaacctg cccaacgaga

9541 aggtgctgcc caagcacagc ctgctgtacg agtacttcac cgtgtataac gagctgacca

9601 aagtgaaata cgtgaccgag ggaatgagaa agcccgcctt cctgagcggc gagcagaaaa

9661 aggccatcgt ggacctgctg ttcaagacca accggaaagt gaccgtgaag cagctgaaag

9721 aggactactt caagaaaatc gagtgcttcg actccgtgga aatctccggc gtggaagatc

9781 ggttcaacgc ctccctgggc acataccacg atctgctgaa aattatcaag gacaaggact

9841 tcctggacaa tgaggaaaac gaggacattc tggaagatat cgtgctgacc ctgacactgt

9901 ttgaggacag agagatgatc gaggaacggc tgaaaaccta tgcccacctg ttcgacgaca

9961 aagtgatgaa gcagctgaag cggcggagat acaccggctg gggcaggctg agccggaagc

10021 tgatcaacgg catccgggac aagcagtccg gcaagacaat cctggatttc ctgaagtccg

10081 acggcttcgc caacagaaac ttcatgcagc tgatccacga cgacagcctg acctttaaag

10141 aggacatcca gaaagcccag gtgtccggcc agggcgatag cctgcacgag cacattgcca

10201 atctggccgg cagccccgcc attaagaagg gcatcctgca gacagtgaag gtggtggacg

10261 agctcgtgaa agtgatgggc cggcacaagc ccgagaacat cgtgatcgaa atggccagag

10321 agaaccagac cacccagaag ggacagaaga acagccgcga gagaatgaag cggatcgaag

10381 agggcatcaa agagctgggc agccagatcc tgaaagaaca ccccgtggaa aacacccagc

10441 tgcagaacga gaagctgtac ctgtactacc tgcagaatgg gcgggatatg tacgtggacc

10501 aggaactgga catcaaccgg ctgtccgact acgatgtgga ccatatcgtg cctcagagct

10561 ttctgaagga cgactccatc gacaacaagg tgctgaccag aagcgacaag aaccggggca

10621 agagcgacaa cgtgccctcc gaagaggtcg tgaagaagat gaagaactac tggcggcagc

10681 tgctgaacgc caagctgatt acccagagaa agttcgacaa tctgaccaag gccgagagag

10741 gcggcctgag cgaactggat aaggccggct tcatcaagag acagctggtg gaaacccggc

10801 agatcacaaa gcacgtggca cagatcctgg actcccggat gaacactaag tacgacgaga

10861 atgacaagct gatccgggaa gtgaaagtga tcaccctgaa gtccaagctg gtgtccgatt

10921 tccggaagga tttccagttt tacaaagtgc gcgagatcaa caactaccac cacgcccacg

10981 acgcctacct gaacgccgtc gtgggaaccg ccctgatcaa aaagtaccct aagctggaaa

11041 gcgagttcgt gtacggcgac tacaaggtgt acgacgtgcg gaagatgatc gccaagagcg

11101 agcaggaaat cggcaaggct accgccaagt acttcttcta cagcaacatc atgaactttt

11161 tcaagaccga gattaccctg gccaacggcg agatccggaa gcggcctctg atcgagacaa

11221 acggcgaaac cggggagatc gtgtgggata agggccggga ttttgccacc gtgcggaaag

11281 tgctgagcat gccccaagtg aatatcgtga aaaagaccga ggtgcagaca ggcggcttca

11341 gcaaagagtc tatcctgccc aagaggaaca gcgataagct gatcgccaga aagaaggact

11401 gggaccctaa gaagtacggc ggcttcgaca gccccaccgt ggcctattct gtgctggtgg

11461 tggccaaagt ggaaaagggc aagtccaaga aactgaagag tgtgaaagag ctgctgggga

11521 tcaccatcat ggaaagaagc agcttcgaga agaatcccat cgactttctg gaagccaagg

11581 gctacaaaga agtgaaaaag gacctgatca tcaagctgcc taagtactcc ctgttcgagc

11641 tggaaaacgg ccggaagaga atgctggcct ctgccggcga actgcagaag ggaaacgaac

11701 tggccctgcc ctccaaatat gtgaacttcc tgtacctggc cagccactat gagaagctga

11761 agggctcccc cgaggataat gagcagaaac agctgtttgt ggaacagcac aagcactacc

11821 tggacgagat catcgagcag atcagcgagt tctccaagag agtgatcctg gccgacgcta

11881 atctggacaa agtgctgtcc gcctacaaca agcaccggga taagcccatc agagagcagg

11941 ccgagaatat catccacctg tttaccctga ccaatctggg agcccctgcc gccttcaagt

12001 actttgacac caccatcgac cggaagaggt acaccagcac caaagaggtg ctggacgcca

12061 ccctgatcca ccagagcatc accggcctgt acgagacacg gatcgacctg tctcagctgg

12121 gaggcgacaa aaggccggcg gccacgaaaa aggccggcca ggcaaaaaag aaaaagtaag

12181 gatccagaga ctcttatcaa gaatcccatc tcttgcttgc ttttttttgt tgcttccctt

12241 tgatagggtt tgtttttctt gtttcagtga ctttctatgt taaaagataa tgtcagtaaa

12301 aggatttggt tttctattat tctgaatcga ttacggaaga ttcttgctta attccaatct

12361 atacaagtat cgtgaaataa tgaccgttta tgtggtaccg agctcgaatt cgtaatcatg

12421 gtcatagctg tttcctgtgt gaaattgtta tccgctcaca attccacaca acatacgagc

12481 cggaagcata aagtgtaaag cctggggtgc ctaatgagtg agctaactca cattaattgc

12541 gttgcgctca ctgcccgctt tccagtcggg aaacctgtcg tgccagctgc attaatgaat

12601 cggccaacgc gcggggagag gcggtttgcg tattggctag agcagcttgc caacatggtg

12661 gagcacgaca ctctcgtcta ctccaagaat atcaaagata cagtctcaga agaccaaagg

12721 gctattgaga cttttcaaca aagggtaata tcgggaaacc tcctcggatt ccattgccca

12781 gctatctgtc acttcatcaa aaggacagta gaaaaggaag gtggcaccta caaatgccat

12841 cattgcgata aaggaaaggc tatcgttcaa gatgcctctg ccgacagtgg tcccaaagat

12901 ggacccccac ccacgaggag catcgtggaa aaagaagacg ttccaaccac gtcttcaaag

12961 caagtggatt gatgtgataa catggtggag cacgacactc tcgtctactc caagaatatc

13021 aaagatacag tctcagaaga ccaaagggct attgagactt ttcaacaaag ggtaatatcg

13081 ggaaacctcc tcggattcca ttgcccagct atctgtcact tcatcaaaag gacagtagaa

13141 aaggaaggtg gcacctacaa atgccatcat tgcgataaag gaaaggctat cgttcaagat

13201 gcctctgccg acagtggtcc caaagatgga cccccaccca cgaggagcat cgtggaaaaa

13261 gaagacgttc caaccacgtc ttcaaagcaa gtggattgat gtgatatctc cactgacgta

13321 agggatgacg cacaatccca ctatccttcg caagaccttc ctctatataa ggaagttcat

13381 ttcatttgga gaggacacgc tgaaatcacc agtctctctc tacaaatcta tctctctcga

13441 gatggtgagc aagggcgagg aggataacat ggccatcatc aaggagttca tgcgcttcaa

13501 ggtgcacatg gagggctccg tgaacggcca cgagttcgag atcgagggcg agggcgaggg

13561 ccgcccctac gagggcaccc agaccgccaa gctgaaggtg accaagggtg gccccctgcc

13621 cttcgcctgg gacatcctgt cccctcagtt catgtacggc tccaaggcct acgtgaagca

13681 ccccgccgac atccccgact acttgaagct gtccttcccc gagggcttca agtgggagcg

13741 cgtgatgaac ttcgaggacg gcggcgtggt gaccgtgacc caggactcct ccctgcagga

13801 cggcgagttc atctacaagg tgaagctgcg cggcaccaac ttcccctccg acggccccgt

13861 aatgcagaag aagaccatgg gctgggaggc ctcctccgag cggatgtacc ccgaggacgg

13921 cgccctgaag ggcgagatca agcagaggct gaagctgaag gacggcggcc actacgacgc

13981 tgaggtcaag accacctaca aggccaagaa gcccgtgcag ctgcccggcg cctacaacgt

14041 caacatcaag ttggacatca cctcccacaa cgaggactac accatcgtgg aacagtacga

14101 acgcgccgag ggccgccact ccaccggcgg catggacgag ctgtacaagt agctcgagtt

14161 tctccataat aatgtg

//
