# Supplementary material for: A Genotype‐Independent Transformation and Gene‐Editing System for Populus
Source: Plant Biotechnol J. 2025 Sep 13;24(2):520–2. doi: 10.1111/pbi.70364 (PMC12906817; doi:10.1111/pbi.70364)
Supplement: Supplementary file 2 — Figure S1: Root induction in a representative Populus genotype. Figure S2: EGFP expression in newly generated roots of various Populus species. Figure S3: Shoot regeneration from transgenic roots in a representative Populus genotype. Figure S4: Construction of the PDS‐targeting knockout vector for Populus. Figure S5: Detection of single nucleotide polymorphisms at the T2 sgRNA target site in the PDS gene. Figure S6: Gene editing and sequencing analysis of the Populus PDS gene. [file PBI-24-520-s003.docx]

**A Genotype-independent Transformation and Gene-editing System for *Populus***

**Supporting Information**


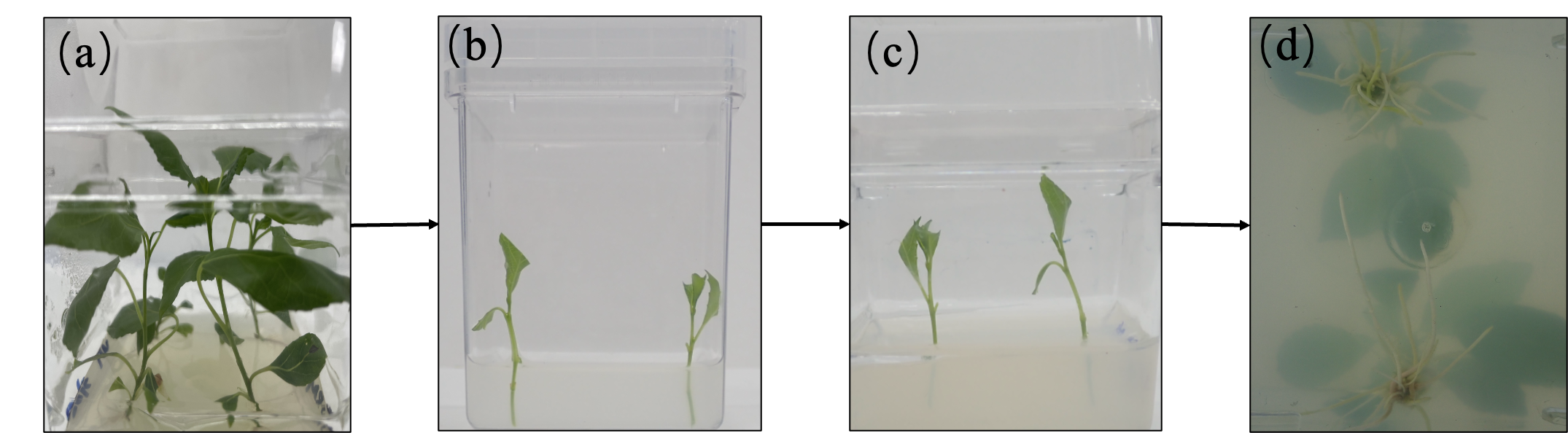


**Figure S1**. Root induction in a representative *Populus* genotype. (a) Two-week-old *P. deltoides × P. euramericana* ‘Nanlin 895’ tissue-cultured plantlets were selected. (b) Stem segments with apical buds were prepared and infected with *Rhizobium rhizogenes* K599. (c) Infected explants were co-cultivated, followed by thorough washing and transfer to hormone- and sugar-free medium to suppress bacterial overgrowth. (d) Roots formed after 30 days.

**Figure S2**. EGFP expression in newly generated roots of various *Populus* species. Fluorescence microscopy was used to detect EGFP expression in newly formed roots after 30 days of induction. EGFP signals were observed at an excitation wavelength of 488 nm and emission at 510 nm. In each image, the upper root is wild-type, and the lower root is fluorescence positive. Scale bar: 2 mm.


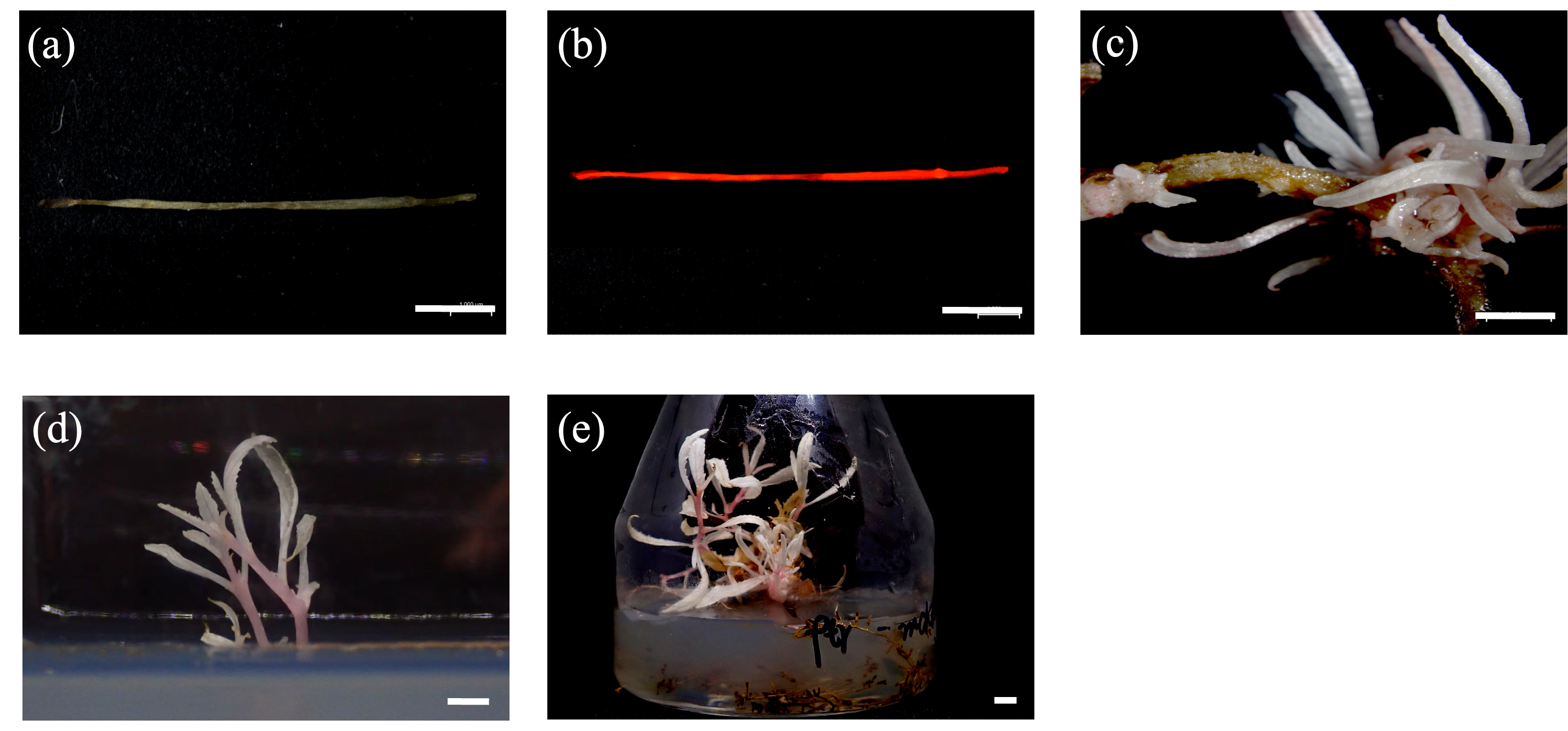


**Figure S3**. Shoot regeneration from transgenic roots in a representative *Populus* genotype. (a, b) mCherry-positive roots of *P. trichocarpa* were used as explants. (c) Transgenic roots were cultured on shoot regeneration medium containing thidiazuron. The construct targeted the *PDS* gene via sgRNA. Albino shoots, resulting from *PDS* knockout. (d, e) Regenerated shoots were transferred to rooting medium and developed into complete plantlets, completing the regeneration cycle. Scale bar: 2 mm.


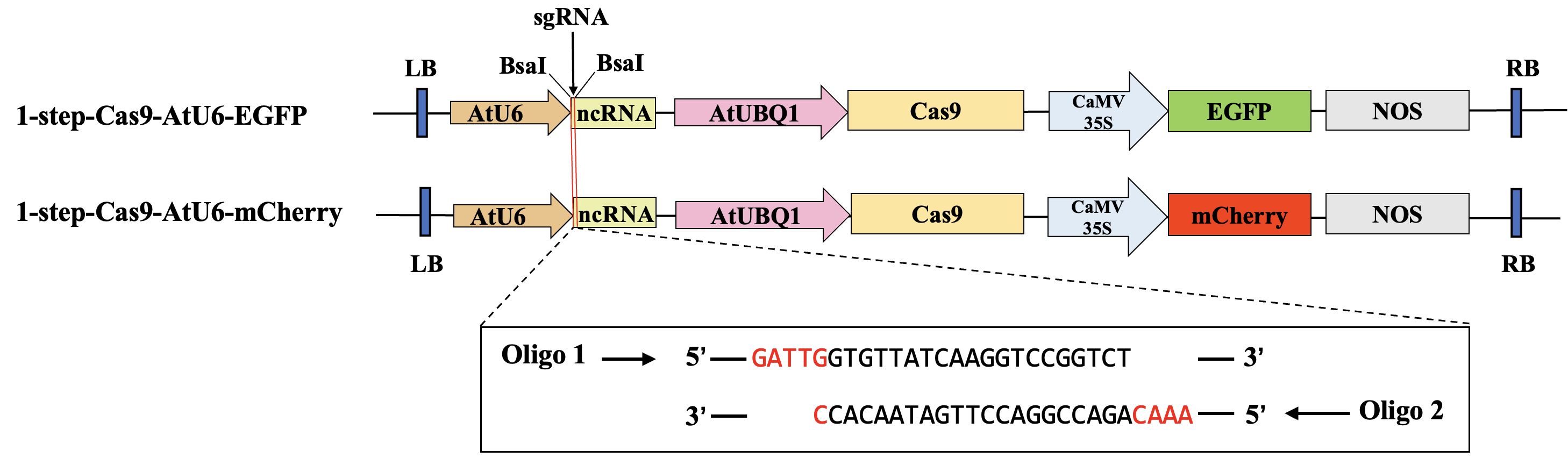


**Figure S4**. Construction of the *PDS*-targeting knockout vector for *Populus*. The *1-step-Cas9-AtU6-EGFP/mCherry* plasmid was digested with *BsaI* to generate sticky ends. sgRNA primers were designed with complementary overhangs: the forward primer (Oligo 1) had a 5′-GATT overhang, and the reverse primer (Oligo 2) had a 5′-AAAC overhang, with an additional cytosine (C) added to the 3′-end of sgRNA. The fragments were then ligated into the vector using T4 DNA ligase.


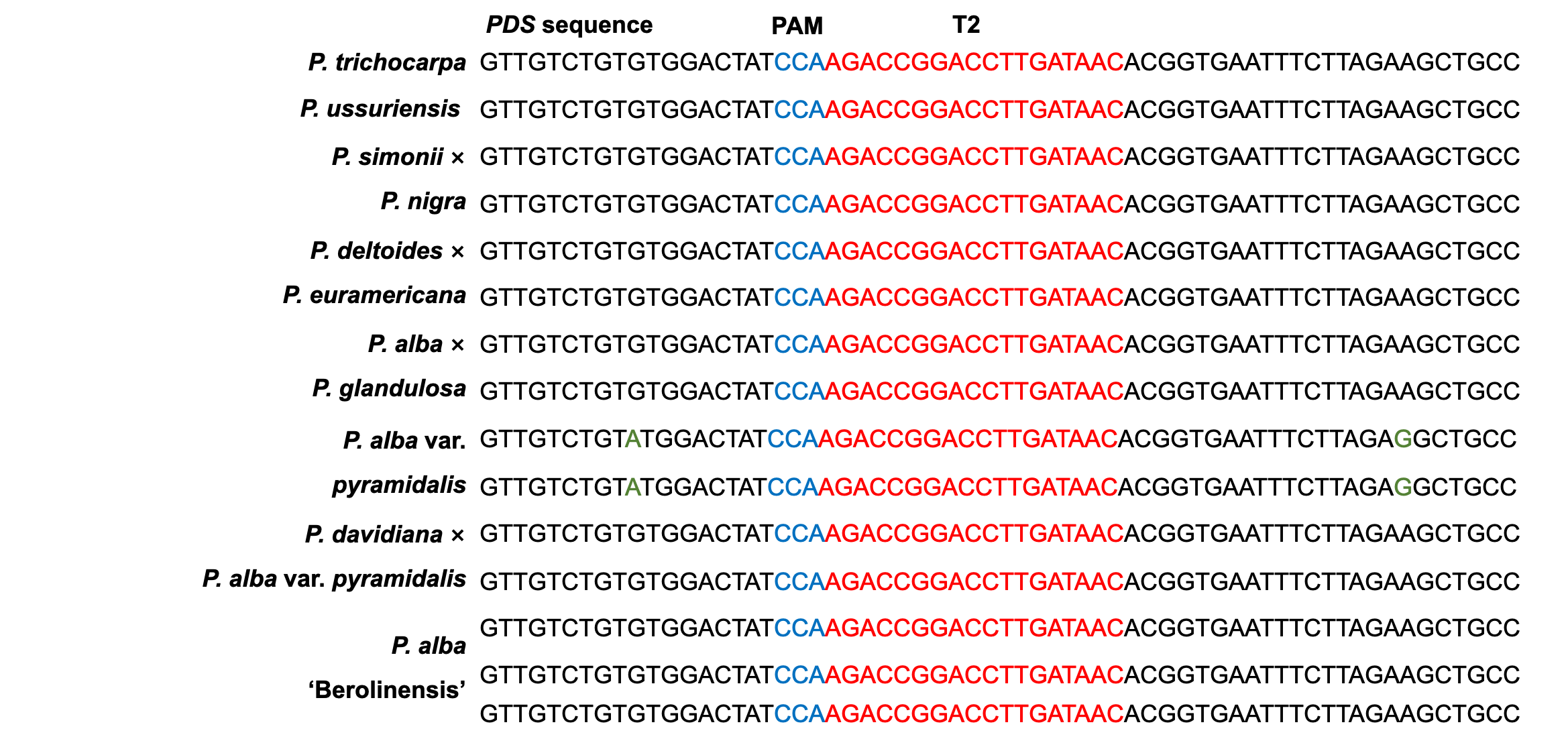


**Figure S5**. Detection of single nucleotide polymorphisms at the T2 sgRNA target site in the *PDS* gene. Single nucleotide polymorphisms (SNPs) at the T2 sgRNA target site in the *PDS* gene were analyzed across eight poplar species. The *P. trichocarpa* *PDS* gene sequence was used as a reference to assess target specificity and sequence conservation. Specific PCR primers were designed to amplify a 200–400 bp genomic region flanking the T2 site. Genomic DNA was extracted, and PCR amplification was performed using high-fidelity DNA polymerase, followed by electrophoresis and sequencing. Sequence alignment revealed that the T2 target region was highly conserved across all tested species, with no SNPs detected. Based on this conserved region, the *1-step-Cas9-AtU6-T2-KO-PDS-EGFP/mCherry* vector is suitable for *PDS* gene knockout in diverse poplar species.

**Figure S6**. Gene editing and sequencing analysis of the *Populus* *PDS* gene. To evaluate the editing efficiency of the CRISPR/Cas9 system targeting *PDS*, genomic DNA was extracted from transgenic roots and albino shoots. The T2 target site region was amplified using PCR and subjected to sequencing analysis. Sequence alignment with the wild-type *PDS* gene revealed that approximately 85% of editing events occurred at the 3rd to 4th nucleotide positions from the 3′-end of the T2 target site. Three major types of mutations were detected: 2- and 1-bp deletions and 1-bp insertions. These mutations introduced frameshifts that resulted in premature termination codons, leading to disrupted *PDS* gene function and the observed albino phenotype due to impaired photosynthetic pigment biosynthesis.
